# Do it yourself: 3D-printed miniature CDC trap for adult mosquito (Diptera: Culicidae) surveillance

**DOI:** 10.1371/journal.pntd.0011899

**Published:** 2024-01-10

**Authors:** Christopher S. Bibbs, Nadja Reissen, M. Andrew Dewsnup, R. Bradley Sorensen, Ary Faraji, Gregory S. White

**Affiliations:** Salt Lake City Mosquito Abatement District, Salt Lake City, UT, United States of America; Liverpool School of Tropical Medicine, UNITED KINGDOM

## Abstract

The central component of mosquito and vector surveillance programs globally is the adult mosquito trap, which is intended to collect host-seeking mosquitoes. The miniature CDC trap is a widely distributed trap style in part due to its relative affordability and compact nature. Despite already being a simple trap, in-house production methods, such as 3D printing, could improve the accessibility of the CDC trap by eliminating some of the supply chain variables. We present here several trials with the Salt Lake City (SLC) trap, a three-dimensional (3D) printed trap design. Functional assessments were made on secondary components and found no statistically significant differences when comparing CO_2_ line height (above vs. below fan), battery types (sealed lead acid vs. USB battery pack), and trap body collection shape (funnel body vs. simple/straight body). The SLC trap was compared directly to a commercial equivalent, the ABC trap, with comparative assessment on species diversity and evenness in collections and found to be statistically equivalent on all metrics. Methods also detail an accompanying optional transport system for a pressurized CO_2_/regulator set-up, should a practitioner elect not to use dry ice. Our final design is presented here with the publicly published stereolithography (STL) files and a detailed outline of the transport container system. Alternative models are available for in-house manufacture of mosquito traps, and we contribute these designs in an effort to stimulate further growth in vector surveillance.

## Author summary

Mosquitoes are among of the most commonly encountered public health pests when discussing zoonotic infectious diseases. Several specific tools exist to collect host-seeking mosquitoes from the field. The miniature CDC trap is a widely distributed, compact trap type that is best used when baited with carbon dioxide, such as through dry ice or pressurized gas. Even if the miniature CDC trap is among the more accessible variants of host-seeking mosquito traps, there can still be barriers to entry for small/local programs needing to trap, learn about, and make management decisions against their local mosquito species. To facilitate this, the Salt Lake City Mosquito Abatement District (SLCMAD) developed a blueprint for 3D printing your own mini-CDC trap and validated its use against commercial equivalents. If a 3D printer is already available through a nearby institution (University, research institute, WHO centers, etc.), then the total cost without the dry ice is ~$4 USD (at the time of writing) to print this trap, as compared to commercially supplied models being ~$200 USD or more. The SLCMAD will continue to improve on the design specifications to require fewer materials and design plans are available publicly without cost at ThingiVerse under the SLCMAD page.

## Introduction

Mosquito abatement programs rely on the information gathered through vector surveillance networks as the justification and support for strategies and interventions performed in the field [1–6]. The core nature of adult mosquito surveillance is critical across the globe [1,7], when dealing with nuisance mosquito populations [1,4,8,9] and outbreaks of mosquito-borne pathogens [10]. Even when focusing only on adult mosquito surveillance, logistical questions can plague program managers: What mosquitoes are being targeted [1,7]; how much sampling is needed to understand population density and longevity [2,4,7,9]; what is the ideal land area needed for monitoring [1,5,7,8]; are chosen traps reliable in the face of environmental conditions, excessive user hours, and possible disruption and sabotage by animals and people [8,11,12]; and what is the monetary cost for the desired surveillance network?

These fundamental questions initially led to the creation of the miniature CDC light trap (CDC LT), which has since become widely used as a relatively cheap and compact trap option allowing mosquito surveillance to be conducted across a wide range of environments in a short amount of time [2,4,5,6]. The CDC light trap design is already simple, but when starting or maintaining a surveillance network of 30+ traps set and collected weekly, a team may still struggle to get enough traps and needed components into remote or resource poor locations. Commercial suppliers can help, but they too can be a source or bottleneck, with the now defunct BioQuip Products being a key example. However, rapid technological growth of on-site-manufacturing, such as laser cutting and three-dimensional (3D) printing, have made constructing mosquito traps easier for organizations of all sizes. New fabrication approaches have yielded rapid, affordable, modular, and more accessible trap models [11,13,14].

Low-cost traps are an integral component in this overarching goal to make surveillance realistic for small budgets or isolated programs. The goal is to reduce cost when establishing new surveillance programs, increase availability, and to simplify repairing and replacing trap components. In this paper, we detail our construction of a 3D-printed vector surveillance trap, as well as the development of field containers that hold all required trap components. We wanted the completed trap and container to be reasonably rugged against elements [8,11,12], the performance to be comparable to commercial CDC-style traps [2,4,5], require low maintenance, and ideally be easy to handle and transport in large numbers [2,8].

## Materials and methods

**Fig 1:**
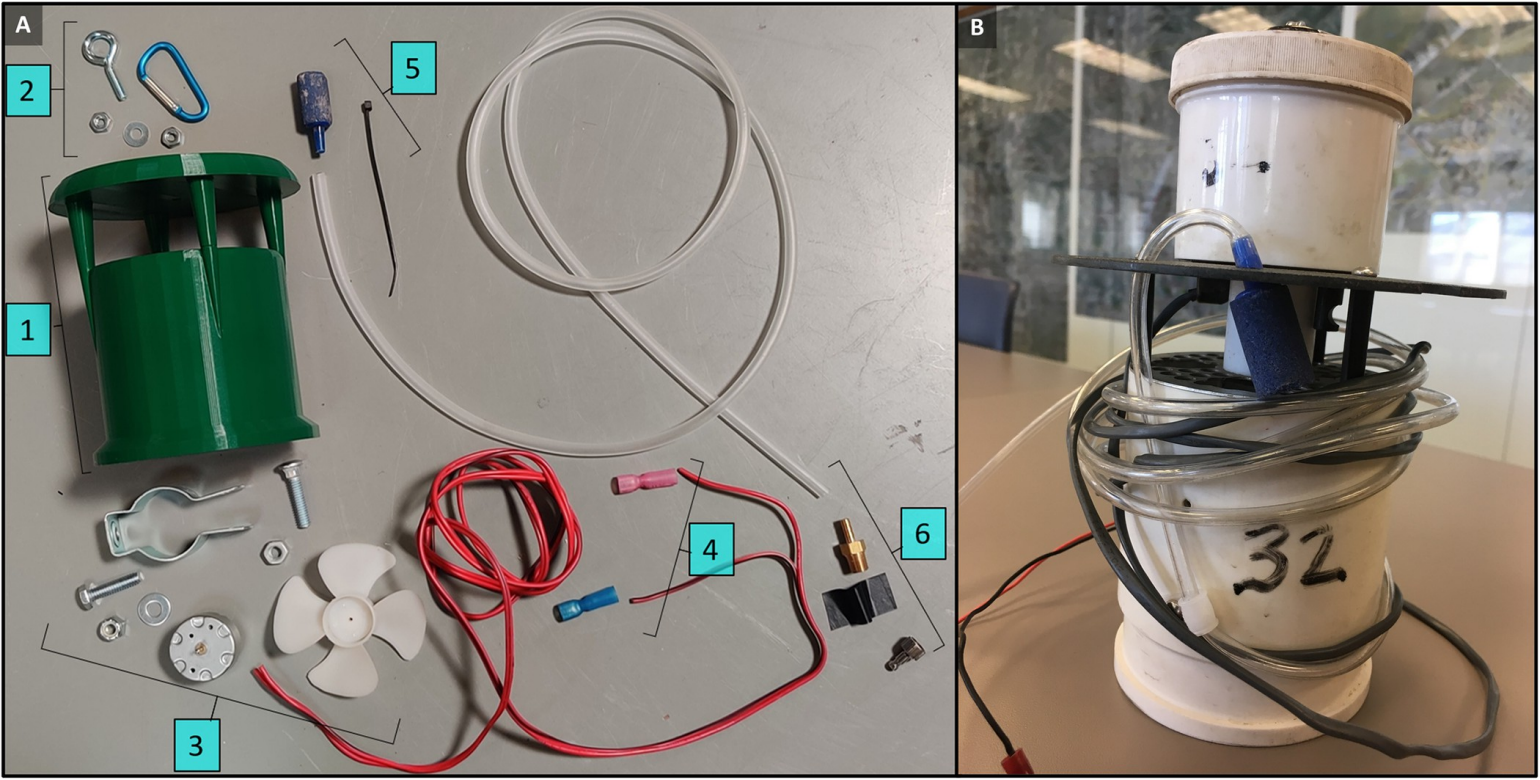
A) Parts layout for the Salt Lake City (SLC) trap: [1]; eye-screws and clips [2]; #2 conduit hanger and a 6-v variable speed motor and 7.5-cm (3 in) propeller; 18-guage copper wire and battery clips [4]; 0.48 cm (3/16 in) tubing with airstone for CO_2_; and push-to-connect fitting to attach to a CO_2_ source of choice. B) Commercial ABC trap, distributed by Clarke Mosquito Control (St. Charles, IL).

A prototype trap design, referred to as the SLC trap, was developed from a variety of exploratory model tests that are found in S1 Fig. Essentially, the prototype is a 1-piece tubular trap body with a rain guard (Fig 1A). A 6-volt variable speed motor (RF-500TB-14415-R-J/32, Nichibo Taiwan Corp., Taipei, Taiwan) was equipped with a 4-blade, 7.5-cm propeller (Item #3C078RSHC1, Thorgren Tool & Molding, Inc., Valparaiso, IN) and secured inside the trap body with #2 conduit hanger (#2 ACC Conduit Hanger, Halex/Scott Fetzer Co, Cleveland, OH). All trap models can be operated using 18-guage, Class 2 copper wire (16-strand dual conductor/182C-200SR, MaxBrite LED Lighting Tech LLC, San Jose, CA). All 3D-printed components were fabricated from PLA plastic filament extruded through a 0.6 mm nozzle with set temperature at 210°C (410°F) and 58°C (136°F) bed temperature. The comparison model was the Clarke (or Pacific Biologics, AUS) branded ABC trap, with the light removed (Clarke Mosquito Control, St. Charles, IL), which is primarily composed of polyethylene and PVC plastics to make a trap body and rain guard (Fig 1B).

### Trap evaluation scheme

**Fig 2:**
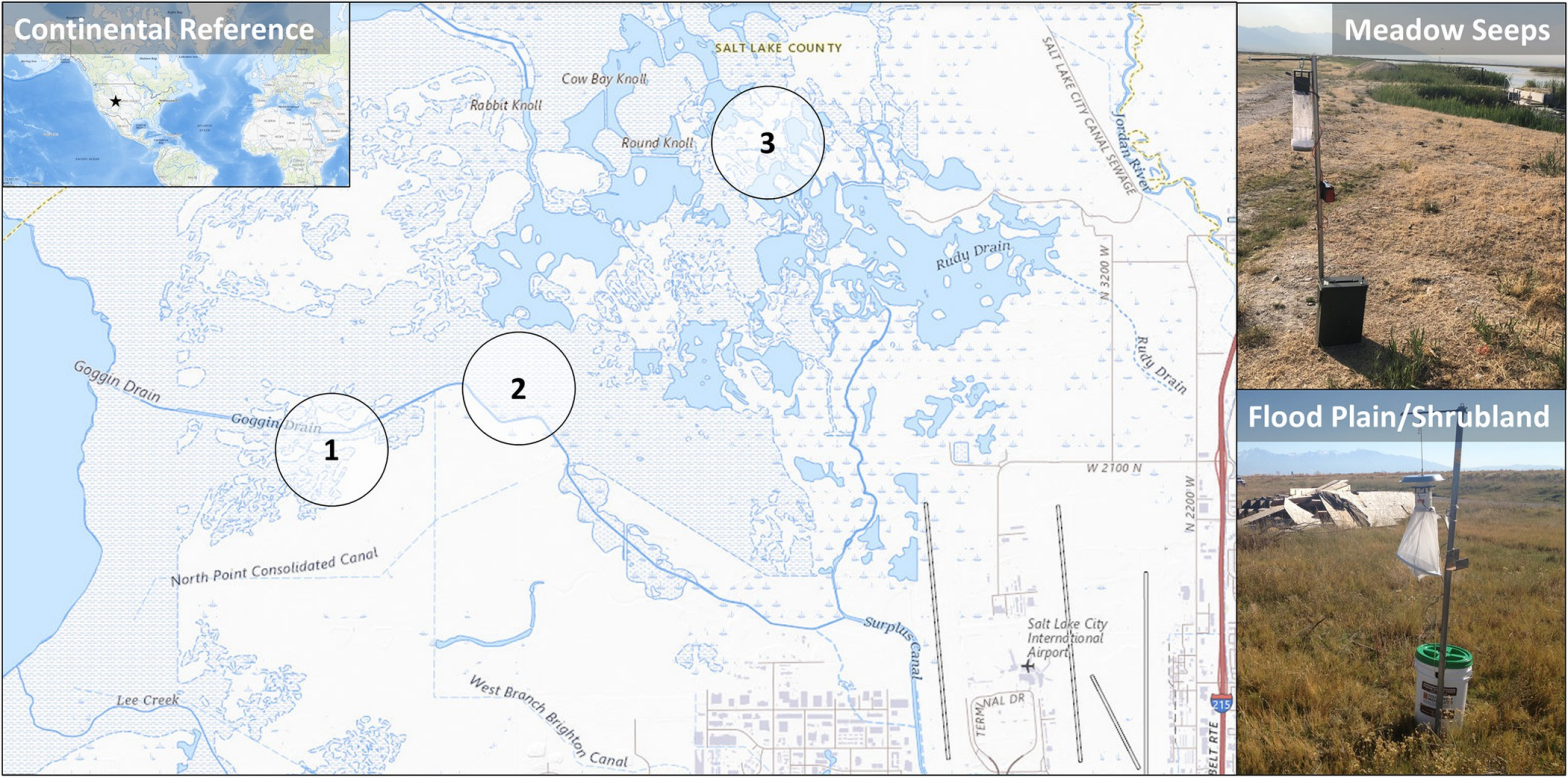
Field sites allocated for testing in the wetland and scrub habitats approaching the Great Salt Lake. Site 1 in the far west of the alkali mud flats outlying the Great Salt Lake. Site 2 near agricultural lands, in flood plans and salt shrub habitat. Site 3 with meadow seeps in freshwater wetlands. The shapefiles for the municipalities, states, country, and hydrography used for the creation of this map were extracted from United States Geological Survey (USGS) (https://basemap.nationalmap.gov/arcgis/rest/services/USGSTNMBlank/MapServer).

Field sites were selected from historical surveillance locations of the Salt Lake City Mosquito Abatement District (SLCMAD) in environments containing a mixture of wetlands and sagebrush. Site 1 was closest to the Great Salt Lake and was composed of muddy shores and salt playas (Fig 2). Site 1 was furthest removed from human influence and in the heart of shorebird and waterfowl habitats in wildlife sanctuary lands. Site 2 (Fig 2) was in the fringes of alkaline mudflats encroached by the Great Salt Lake and composed of a mix of palustrine flood plains and salt desert shrublands that were adjacent to animal agricultural lands. Site 3 was the furthest north location (Fig 2) and was in the freshwater overflow for nearby reservoirs. Site 3 was characterized by saline meadow seeps and saline wetlands and was well outside any human occupied areas. The majority of mosquitoes historically occupying these environments was split between *Aedes dorsalis* (Meigen) and *Culex tarsalis* Coquillett. For SLCMAD, *Cx*. *tarsalis* is a priority vector of West Nile virus. Lights have been previously shown to confound vector *Culex* sp. collections the same general eco-region, in addition to increasing non-target insect numbers [15]. Therefore, all trap nights, regardless of model, excluded the use of a light source.

Surveillance was conducted with 1 kg dry ice according to Sriwichai et al. [5] for trials comparing the SLC trap to the commercial model ABC trap. For experiments that were done for comparing various trap modifications to the SLC trap, the CO_2_ source was from a regulated compressed cylinder at the rate of 250 ml/min. For all trials traps were set for 24 hrs and trap collections were counted and identified with a hybrid camera and microscopy set-up using ImageJ [16]. Species identifications were keyed according to Darsie and Ward [17]. Tests were conducted with different supplies and components to optimize the base model SLC trap. Pairwise comparisons were conducted for SLC trap modifications were conducted exclusively at site 1. Tests were conducted between: CO_2_ dispensed high or low on the trap body (8 replicates); 6-volt sealed lead-acid batteries (PS-6100 6v 12Ah, Power Sonic Co., San Diego, CA) and USB battery packs (PowerCore 20 100mAh, Anker Innovations Co., Ltd, Hunan, China) (10 replicates); a funneled housing option (S2 Fig) (4 replicates). For comparison of the SLC trap to the commercial model ABC trap (positive control), tests were replicated 4 times each at sites 2 and 3 for a total of 8 comparison nights. Whether doing accessory tests or the positive control comparison, both traps were present at the same site but spaced at least 100 m apart. Additional minor tinkering was conducted for transport container types, comparing tool boxes, buckets, and metal ammo cans. These transport containers were investigated for optional use with a pressure regulated CO_2_ cylinder system (moot in the case of dry ice, which can be carried in a separate cooler).

**Fig 3:**
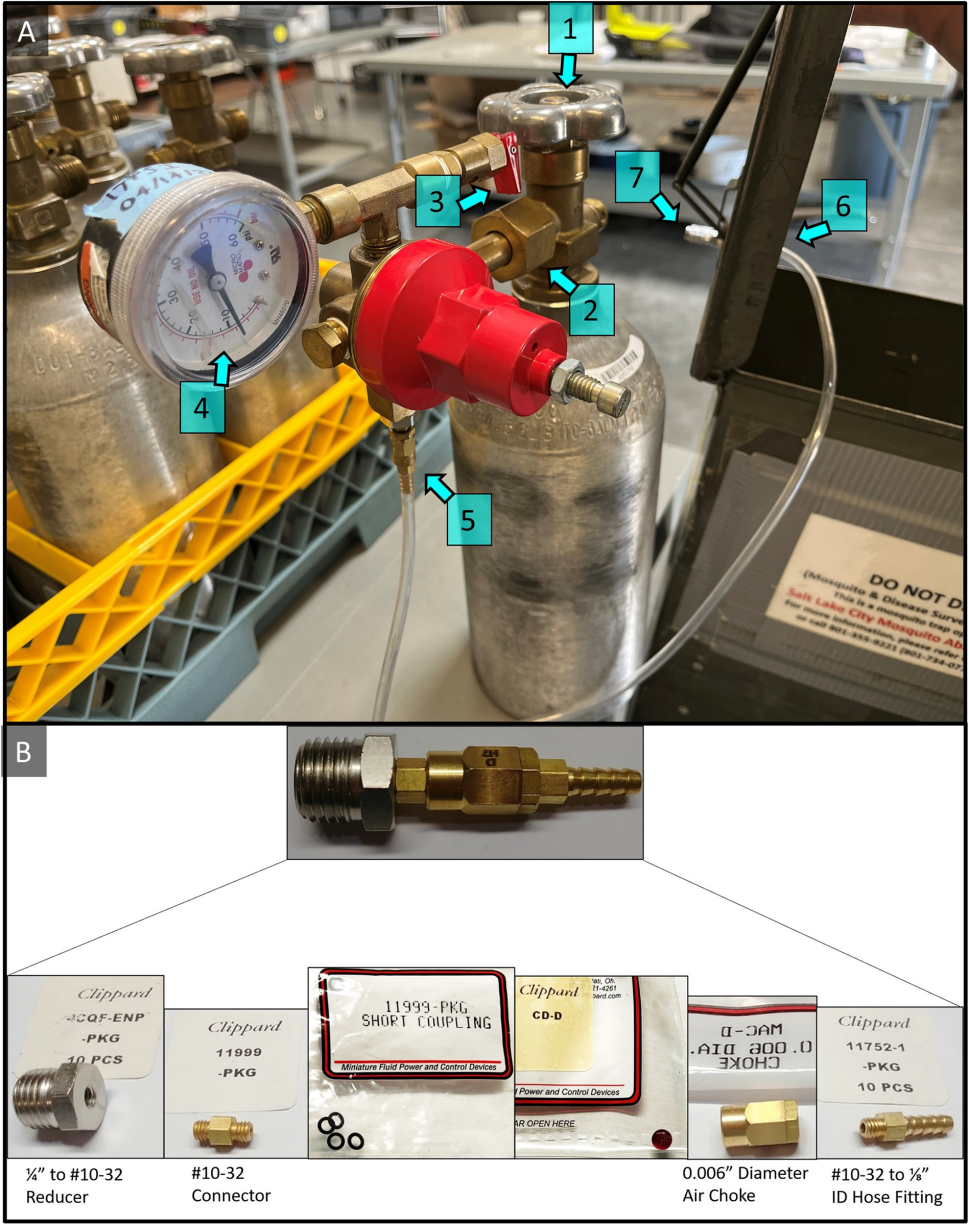
A) CO_2_ cylinder with attached regulator and ammo can; Inspection points: 1) Cylinder valve; 2) Attachment of regulator to cylinder; 3) Bleed valve; 4) Pressure reading; 5) Couplings for CO_2_ line; 6) Connector under ammo can lid; 7) Connector for CO_2_ line to trap. B) Assembly for #5.

**Fig 4:**
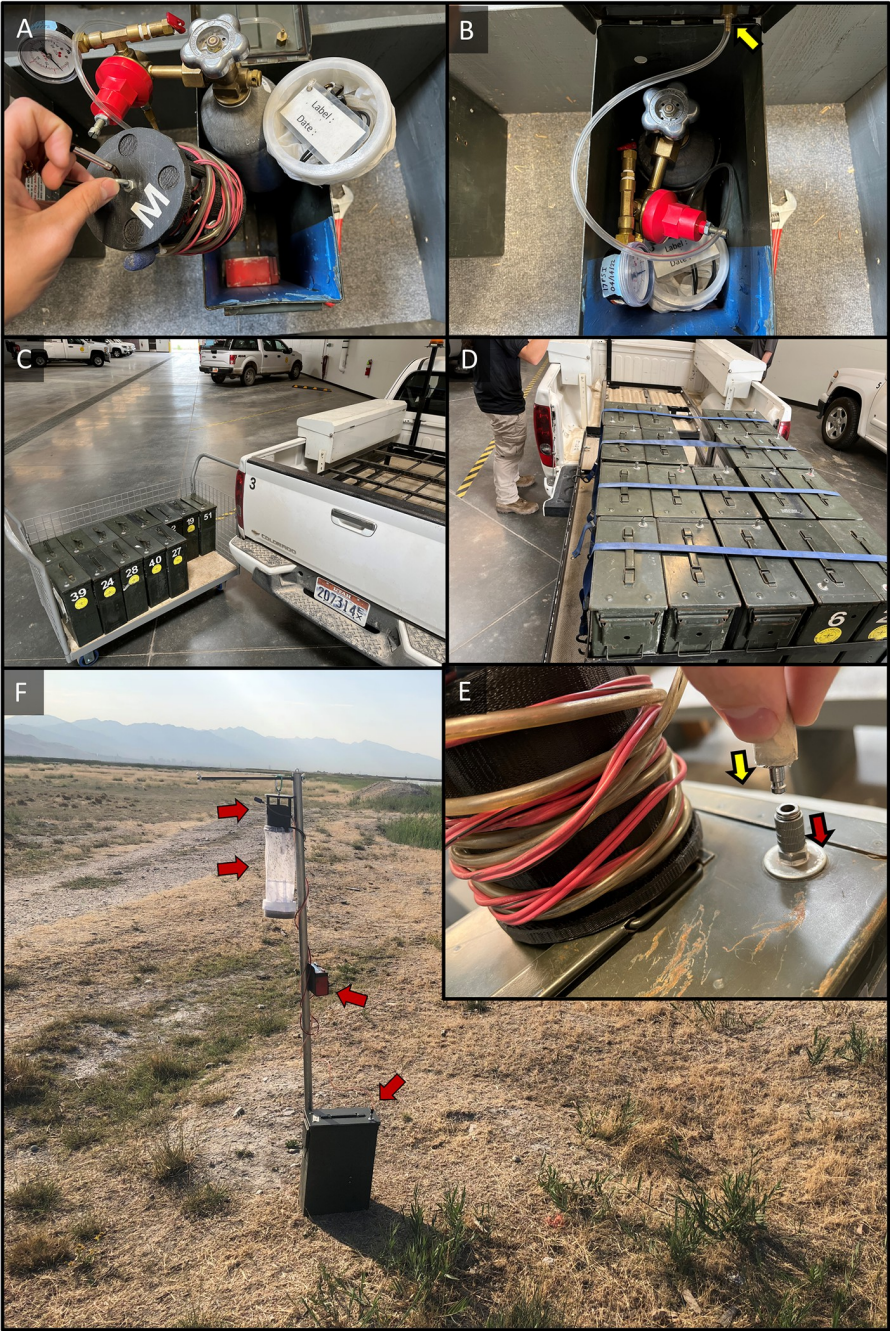
A) Ammo can transport a filled CO_2_ cylinder with regulator, trap fan with airstone, trap net with label, and battery; B) ammo can packed together; C) transport and load; D) squared design fits into racks on a truck bed-slide system; E) trap-to-can CO_2_ connection; F) Fully deployed trap with 6v battery and trap secured to pole and linked with ammo can.

Transport containers were selected based on size, ruggedness and long-term service potential (S2 and S3 Figs). Although it is suitable to embed the battery and CO_2_ source in a toolbox or bucket (S2 Fig), the desire for space efficiency and durability led to selection of a re-purposed metal ammunition can from military surplus (Figs 3 and 4) for operational use at SLCMAD. The resulting final surveillance trap and container design was specially built around using a pressure-regulated CO_2_ cylinder system.

SLCMAD chose to adopt a pressure-regulated CO_2_ cylinder system after the above trials were conducted on trap design. Cylinder regulators were modified with a complex series of small parts to feed the gas safely to a lower CO_2_ line attachment (Fig 3). A “¼ in to #10–32 reducer” (4CQF-ENP-PKG, Clippard Instrument Laboratory, Inc., Cincinnati, OH) was reinforced with PTFE tape and attached directly to the lower regulator. Short coupling O-rings (11999-PKG, Clippard Instrument Laboratory, Inc., Cincinnati, OH) were seated on both sets of threads of a “#10–32 connector” (11999-PKG, Clippard Instrument Laboratory, Inc., Cincinnati, OH), which was then screwed into the reducer. The reducer starts with a “0.0075 in diameter air choke” (CD-C, Clippard Instrument Laboratory, Inc., Cincinnati, OH) that is fitted with a choke disk inside the curved (non-faceted) end of the piece, then screwed onto the remaining threads of another “¼ in to #10–32 reducer”. The faceted end of the air choke is finished by screwing in a “#10–32 to ¼ in ID hose fitting” (11752-1-PKG, Clippard Instrument Laboratory, Inc., Cincinnati, OH) (Fig 3). The CO_2_ line extends off the lower choke assembly (Fig 3) and attaches to the underside of a PA70 ammo can measuring 42 cm × 30.5 cm” × 15.9 cm (B643, 8-Cartridge, 60MM, HE, M888 for M224 Mortar, United States Military Surplus) with another brass “#10–32 to ¼ in ID hose fitting.”

For the transport container itself, the top side of the ammo can lid was fitted with a female push-to-connect piece (Airbrush Quick Release Coupling ⅛-in BSP, Point Zero Ltd., China) that receives the male end (Fig 4) when traps are deployed. To brace the CO_2_ cylinder inside the ammo can, 7–8 cm wide thermoforming plastic sheet (Sekisui Kydex, Bloomsburg, PA) was softened with a heat gun and then molded around the cylinder (S3 Fig). After cooling to form, the sheet was riveted inside the ammo can to create a holster for the cylinder (S3 Fig). The opposite end of the ammo can interior was then reinforced with padding, in this case with duct tape strips, so as to pad the batteries and prevent contact with the battery terminals. (S3 Fig). Once complete, the transport containers receive a 6v battery, filled CO_2_ cylinder, and associated trap with collection net (Fig 4).

### Data analysis

Pairwise comparisons were conducted in R statistical software (v.4.2.1, The R Foundation for Statistical Computing, Vienna, Austria) via RStudio (v. 3.3.0, RStudio PBC, Boston, MA) to generate outputs for each of airstone height (high vs. low), battery (lead-acid vs. USB), funnel body (funnel vs. straight), and commercial comparison (ABC trap vs. SLC trap). Outputs were based on the net abundance of mosquitoes and p values greater than 0.05 were considered non-significant. Species diversity was evaluated for each set of trials. Species richness and evenness were calculated between the SLC trap and the ABC trap. Transport containers were not considered in the trap comparisons, since traps are never confined inside them during field deployment.

## Results

**Fig 5:**
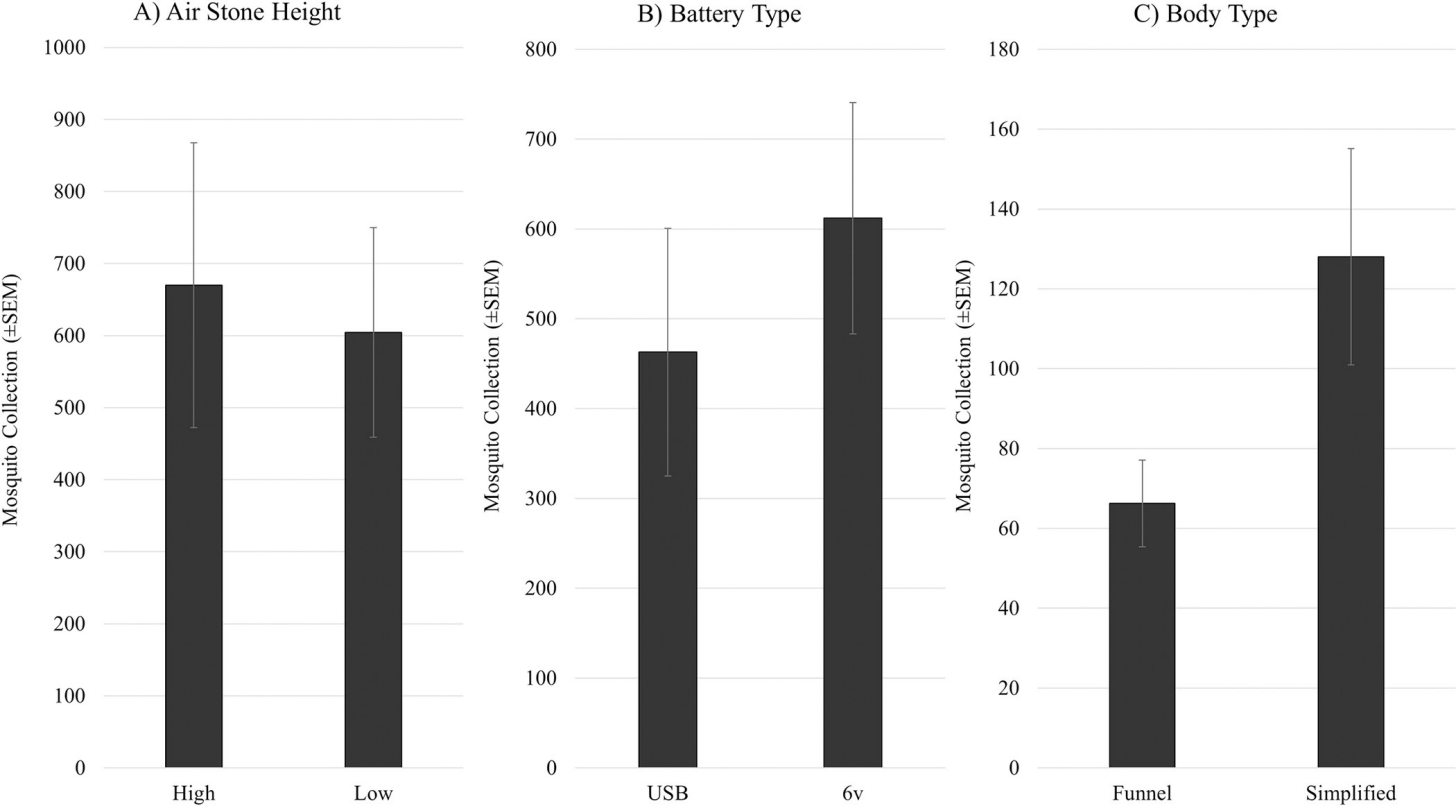
Pairwise test results for airstone/CO2 line positioning (high vs. low), battery options (6v sealed lead acid vs. USB lithium battery pack), and trap body type (funneled vs. straight/simple body). Bar graphs with I-bars as standard error of the mean. All pairings were not significantly different within their group.

For general species diversity, *Aedes dorsalis* (Meigen), *Ae*. *vexans* (Meigen), *Anopheles freeborni* Aitken, *Culex erythrothorax* Dyar, *Cx*. *pipiens* L., *Cx*. *tarsalis* Coquillett, and *Culiseta inornata* (Williston) were collected by every trap variant within each set of trials, resulting in no net difference in species diversity collected across any of the SLC trap modifications or comparison with the ABC trap. For the pairwise comparisons on components of the SLC trap, height of the airstone on the trap body was not significantly different whether lines were mounted low or high on the trap body (Fig 5; t = 0.7241, p = 0.4925). Additionally, battery type did not significantly change overall mosquito collection numbers (Fig 3; t = 1.1943, p = 0.2629). There was a visual trend in mosquito collections being higher in the regular trap body, versus the funnel body, but statistically the differences were not significant (Fig 5; t = 2.2849, p = 0.1065).

**Fig 6:**
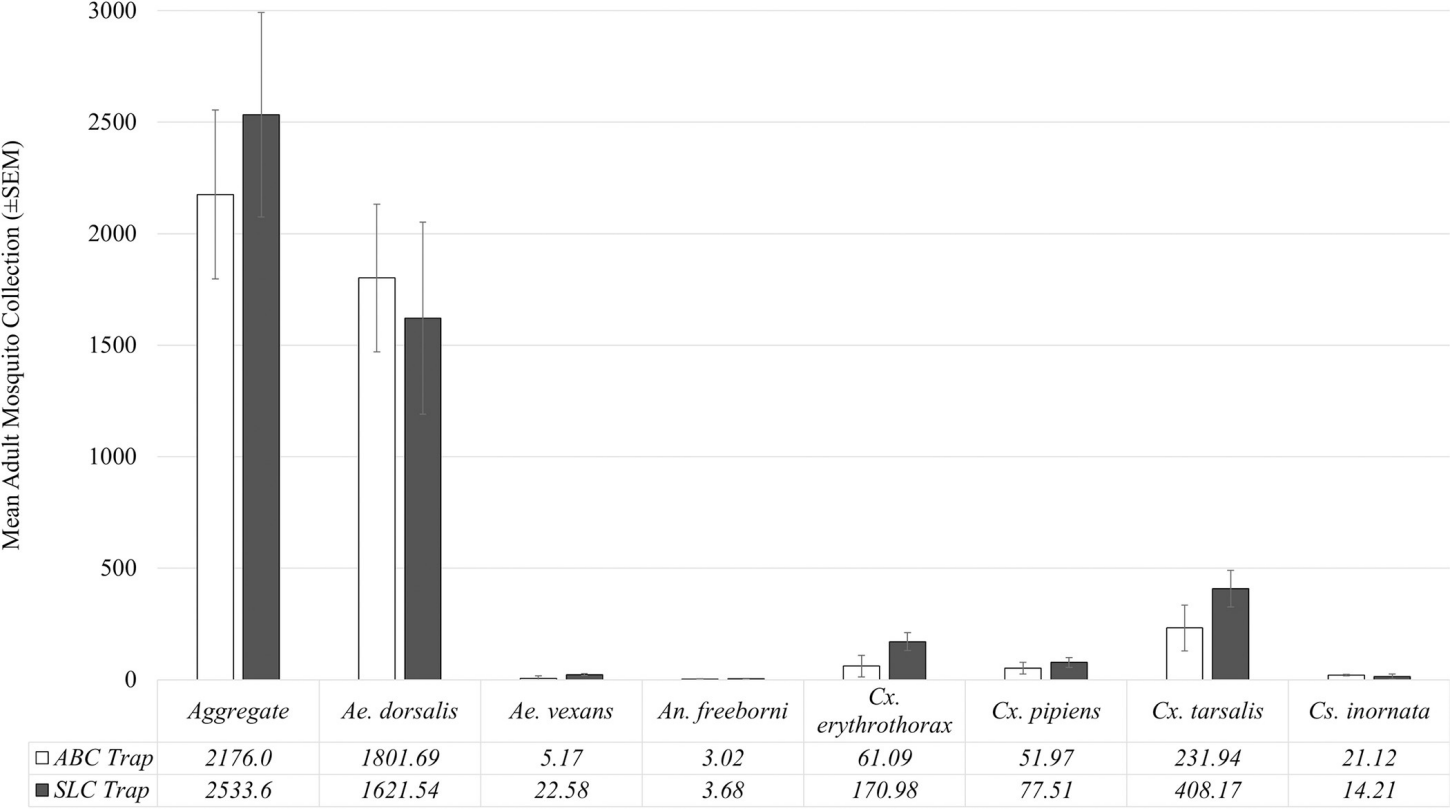
Comparison between the Salt Lake City trap (SLC) and commercial ABC trap (Clarke Mosquito Control, St. Charles, IL) based on adult mosquito collection totals. Bar graphs with I-bars as standard error of the mean. All pairings were not significantly different within their group.

When comparing species richness and evenness between the SLC trap to the ABC trap, values were H = 0.689, E = 0.354 and H = 0.746, E = 0.383, respectively. In agreement with historical data, both *Ae*. dorsalis (81.9%) and *Cx*. *tarsalis* (10.4%) were the most abundant mosquitoes. When listed in order of relative abundance, the remainder of collections were sparse amounts of *Cx*. *erythrothorax* (3.4%), *Cx*. *pipiens* (2.8%), *Cs*. *inornata* (1.1%), *Ae*. *vexans* (0.3%), and *An*. *freeborni* (0.1%). For total mosquito collections, the SLC trap and ABC trap were not significantly different from each other (Fig 6; t = -0.5125, p = 0.624).

## Discussion

The SLC trap design is available as a stereolithography (STL) file on ThingiVerse under SLCMAD (https://www.thingiverse.com/slcmad/designs), which is publicly available to all. Generally, most of the trap amendments in our investigations were not drastically different from each other in mosquito collections, whether by mosquito abundance or species composition. However, the USB battery packs did incorporate a failsafe that de-energized the motors if a lack of adequate power draw was detected. This led to unexpected trap failures, even though the USB batteries in theory would allow longer activity windows for traps. The richness and evenness calculated in our study is typical of the historical data at SLCMAD in that *Ae*. *dorsalis* and *Cx*. *tarsalis* tend to overwhelmingly dominate any trap collections. In the context of this study, there was any meaningful loss of diversity detected between the SLC trap and the commercial ABC trap.

Generally, designs for self-manufacture of mosquito surveillance traps are currently a low published area in literature. A model is presented by Hoshi et al. [13], but may be less rugged to the wear and tear of routine use. Another design is the Multifunctional Mosquito Trap (MMT) developed by Reinbold-Wasson and Reiskind [14]. The MMT is a unique hybrid between gravid and sentinel traps intended to target container-inhabiting *Aedes* sp. in a resource efficient manner. In contrast, our simplified CO_2_ trap design is a traditional miniature CDC trap analog, the latter of which has a broader expected species diversity [2,4,5,15,18]. There is still ample room to grow the concepts of on-site manufacturing (such as through 3D printing). For the few other contributions in this area [13,14], the consensus is to embrace modification and creative problem solving using these tools to improve mosquito surveillance in a cost-effective manner. For example, gravid trap designs [19], downdraft/updraft trap experimentation, or hybrids such as the MMT [14], are all avenues for future investigation.

When expecting seasonal virus activity and nuisance mosquito outbreaks, failing to collect surveillance data can negatively impact public health and the well-being of constituents [11,12]. Surveillance also is generally harmed by having traps that are expensive or difficult to repair, replace, or maintain service life [2,8,20]. The SLC trap was intended as an easy to build model of the mini-CDC trap to help enable low-budget programs to avoid the aforementioned surveillance deficiencies. Mobilizing even 10–20 traps per person can be prohibitive once you consider batteries, lures, and supplies to secure or tether traps in the field [2,8,11,12,21]. We hope that the added ideas for our described storage-transport system (Figs 3, 4, S2 and S3) can help alleviate some of the operational difficulties that accompany surveillance programs.

The transport system itself ties into SLCMAD switching CO_2_ sources from dry ice to a pressure-regulated CO_2_ cylinder system following study using the SLC trap. For the locality in which SLCMAD occurs, the cost of CO_2_ by dry ice was found to be about 20x higher a week than the cost of CO_2_ from pressurized cylinders for the same number of trap nights. Logistically, the purchasing, storing, and trap preparation with CO_2_ from pressurized tanks allowed greater flexibility in work schedules for surveillance technicians throughout the mosquito season. A factor that affects collection numbers is the standardization in CO_2_ flow rate via pressure regulated systems. Dry ice sublimation fluctuates greatly depending on humidity, temperature oscillation, and microclimates [22,23,24]. However, CO_2_ cylinders may be a luxury in some areas, necessitating other CO_2_ sources, such as yeast fermentation containers [25,26,27], as more widely available options.

We would encourage other research groups to also consider making equipment schematics publicly available for low-resource programs. The opportunity to creatively build on those public designs, or pivot into other essential vector control equipment, could be a great long-term benefit for the public health community. The SLC trap can still be improved in many ways to make the trap easier to assemble and repair, better at attracting different vectors, more durable and easier to use with compressed CO_2_. Modifications SLCMAD is currently evaluating for future models include making the rain cover removable, replacing the fan motor mount with a printable bracket, increased print-fill density for impact resistance, a slot to hold the airstone/CO_2_ hose, and optional brackets for mounting a light source. Should a reader visit the public STL on ThingiVerse in the future, the latest model employed by SLCMAD will be available.

## Supporting information

S1 Fig Six different prototypes of traps during initial screening. A) 3-piece base design developed using an entry funnel mounted to a computer case fan, then stacked with a second funnel for connecting a catch net; used for 3 trap designs: a 12-volt case fan (Tornado TD8038H, Vantec Thermal Technologies, Fremont, CA) and measured at 20-kph suction. A 6-volt case fan (Multifan S1 80mm, AC Infinity, Inc., City of Industry, CA) measuring at 12-kph suction was then used for two separate models: “Complex Airstone” containing a 5-mm mineral airstone (Jardin Stone, UXCell Co., Hong Kong, China) on the CO_2_ line (4-mm inner diameter standard aquarium tubing, Penn-Plax, inc., Hauppauge, NY) for dispersing a lure homogenously; and “Pore Dispersal” where the CO_2_ line was fitted directly to the fan. B) The Salt Lake City trap covered in the main manuscript. C) 3D-printed trap design shared by Mosquito Consulting Services based in New Zealand. D) Positive control of the ABC trap (Clarke Mosquito Control, St. Charles, IL). E) Comparison data with a minimum of 4 replicates each and using aggregate adult mosquito collections. Outliers are black points outside the range of the box whiskers. Blue points denote the mean and the central black bar reflects the median.
(TIF)

S2 Fig 1) Rejected design for funnel shaped trap body. 2) Printing errors that occur when the bed and nozzle settings are not correct for your build. 3) Rejected, but suitable, bucket container option for transport. 4) Rejected, but suitable, toolbox container option for transport.
(TIF)

S3 Fig 1) Thermoforming plastic strip cut to a reasonable length to encircle your desired CO_2_ cylinder. 2) Molding the plastic to the desired cylinder and allowing to cool to shape. 3) Riveting holster inside the ammo can and reinforcing the opposite edge with tape to protect batteries during use.
(TIF)
