# Supplementary figures and images for: Do it yourself: 3D-printed miniature CDC trap for adult mosquito (Diptera: Culicidae) surveillance
Source: PLoS Negl Trop Dis. 2024 Jan 10;18(1):e0011899. doi: 10.1371/journal.pntd.0011899 (PMC10805281; doi:10.1371/journal.pntd.0011899)

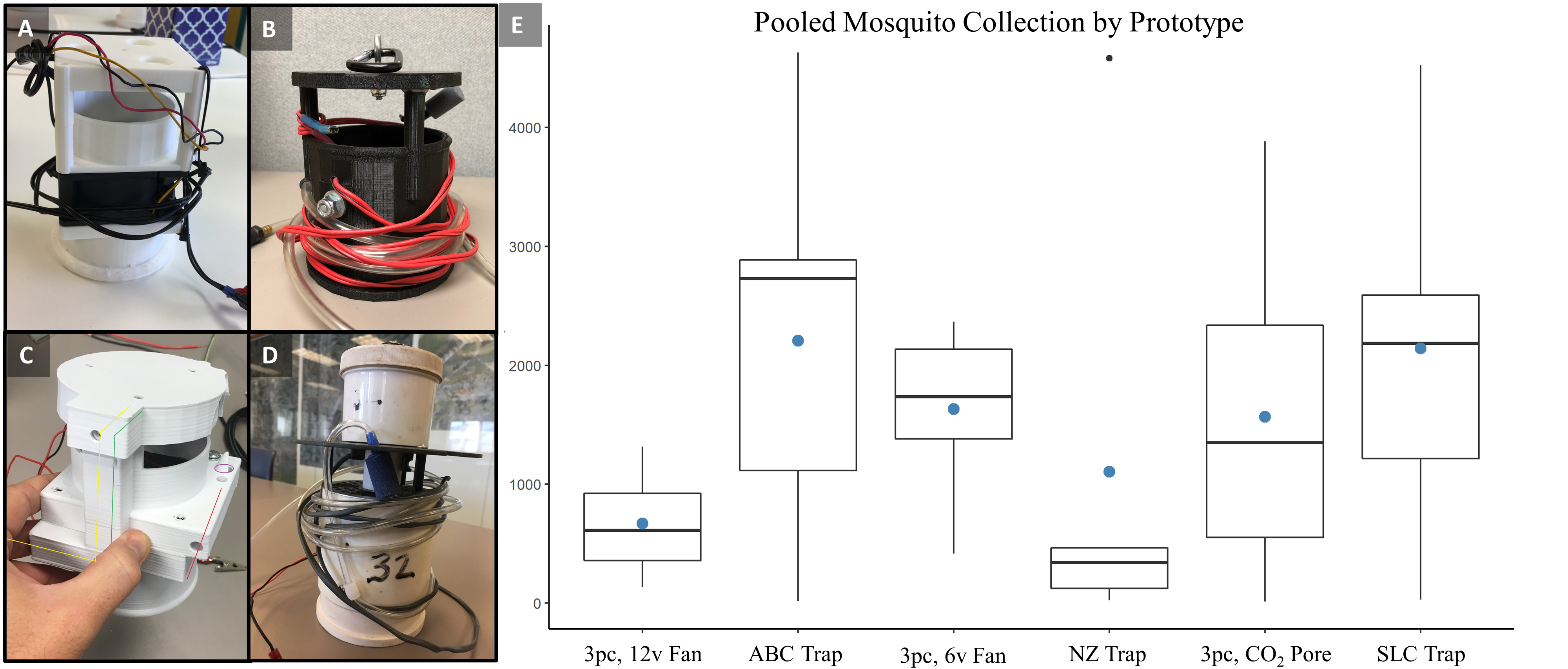

Supplement: S1 Fig — A) 3-piece base design developed using an entry funnel mounted to a computer case fan, then stacked with a second funnel for connecting a catch net; used for 3 trap designs: a 12-volt case fan (Tornado TD8038H, Vantec Thermal Technologies, Fremont, CA) and measured at 20-kph suction. A 6-volt case fan (Multifan S1 80mm, AC Infinity, Inc., City of Industry, CA) measuring at 12-kph suction was then used for two separate models: “Complex Airstone” containing a 5-mm mineral airstone (Jardin Stone, UXCell Co., Hong Kong, China) on the CO2 line (4-mm inner diameter standard aquarium tubing, Penn-Plax, inc., Hauppauge, NY) for dispersing a lure homogenously; and “Pore Dispersal” where the CO2 line was fitted directly to the fan. B) The Salt Lake City trap covered in the main manuscript. C) 3D-printed trap design shared by Mosquito Consulting Services based in New Zealand. D) Positive control of the ABC trap (Clarke Mosquito Control, St. Charles, IL). E) Comparison data with a minimum of 4 replicates each and using aggregate adult mosquito collections. Outliers are black points outside the range of the box whiskers. Blue points denote the mean and the central black bar reflects the median. (TIF) [file pntd.0011899.s001.tif]

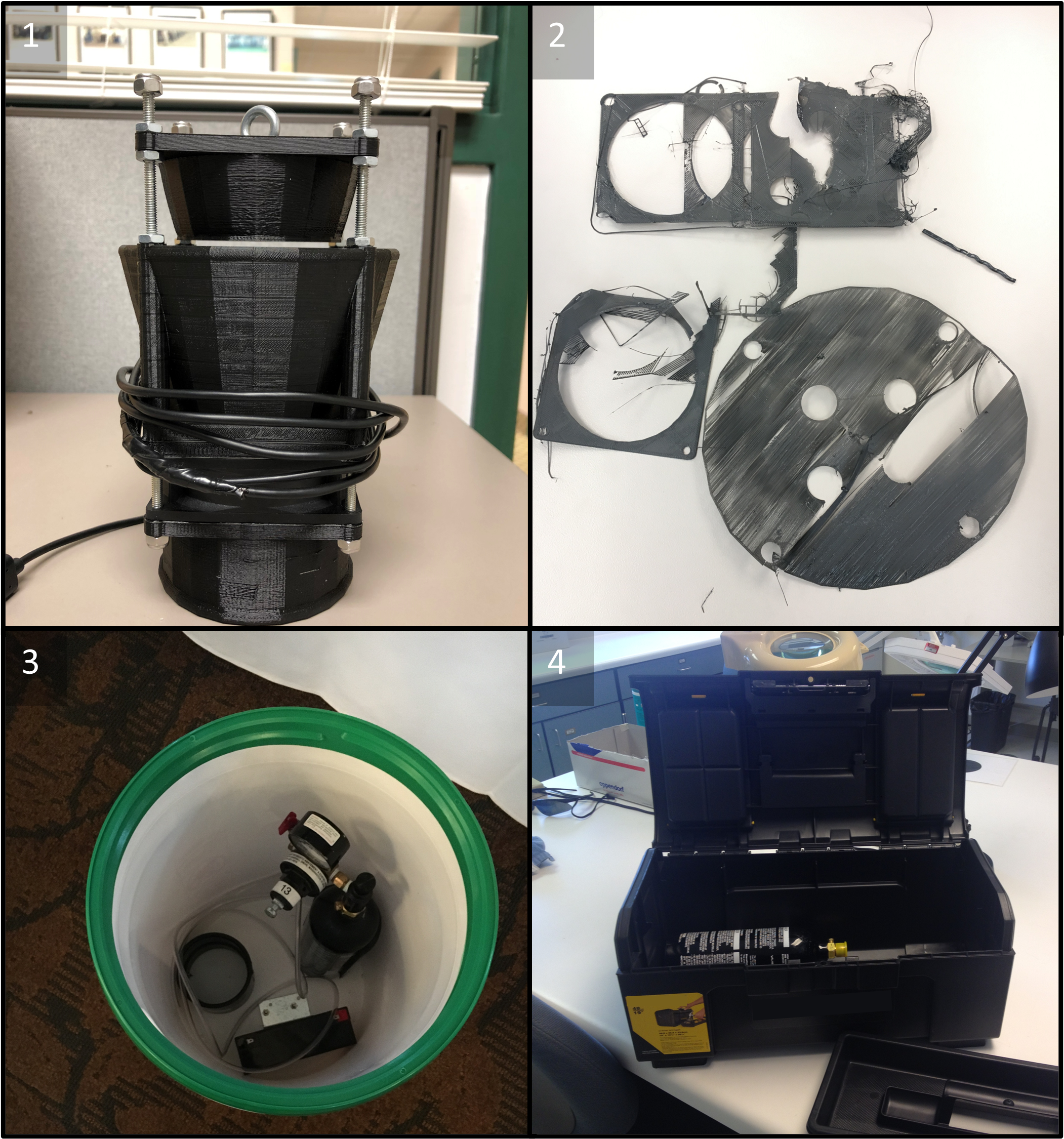

Supplement: S2 Fig — 2) Printing errors that occur when the bed and nozzle settings are not correct for your build. 3) Rejected, but suitable, bucket container option for transport. 4) Rejected, but suitable, toolbox container option for transport. (TIF) [file pntd.0011899.s002.tif]

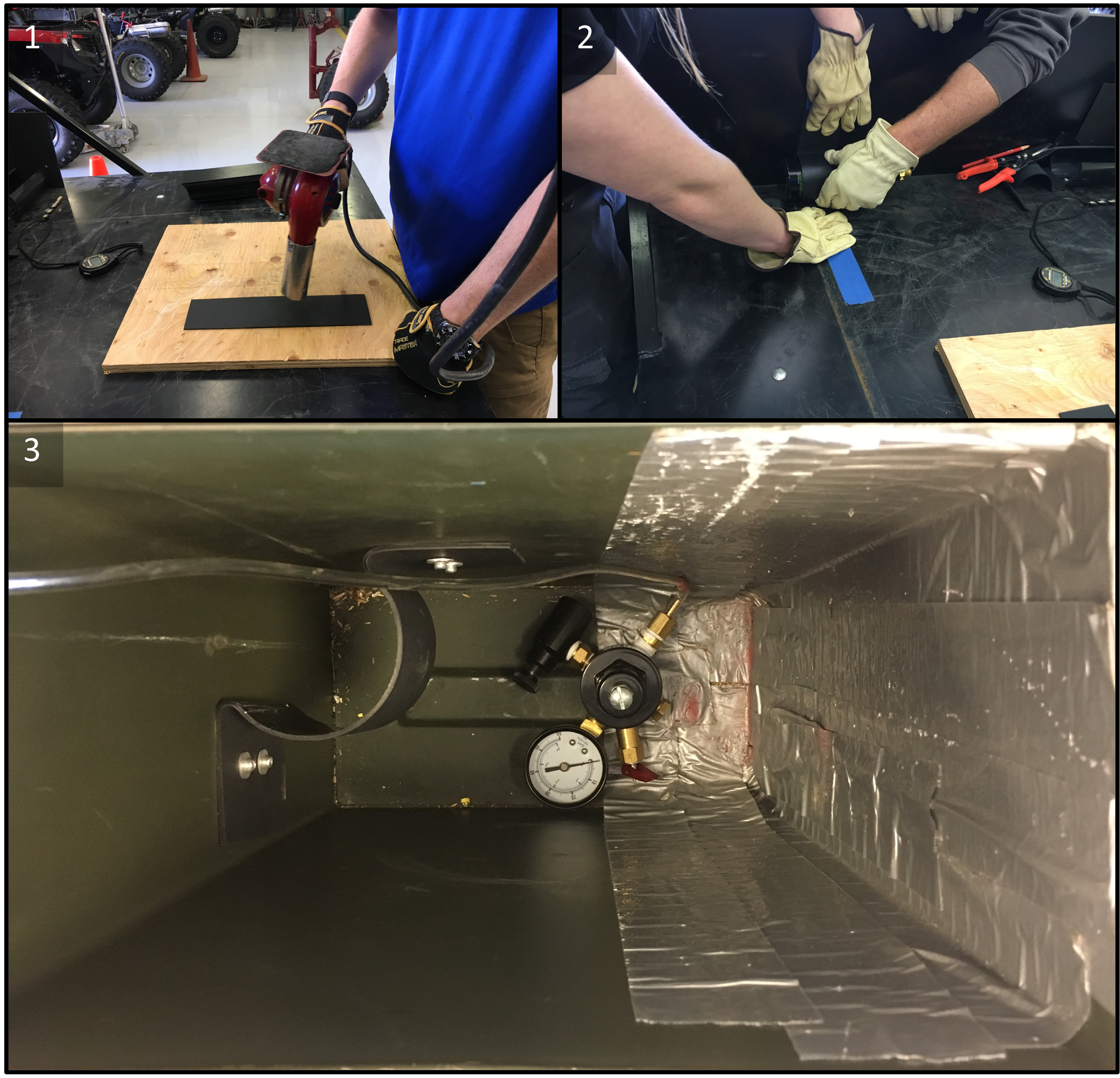

Supplement: S3 Fig — 2) Molding the plastic to the desired cylinder and allowing to cool to shape. 3) Riveting holster inside the ammo can and reinforcing the opposite edge with tape to protect batteries during use. (TIF) [file pntd.0011899.s003.tif]
